# Prosthetic temporomandibular joint reconstruction in a cohort of adolescent females with juvenile idiopathic arthritis

**DOI:** 10.1186/s12969-020-00453-6

**Published:** 2020-09-04

**Authors:** Michael Lypka, Karina Shah, Jordan Jones

**Affiliations:** 1 Division of Plastic and Craniofacial Surgery, Children’s Mercy Hospital, 2401 Gillham Road, Kansas City, MO 64108 USA; 2 University of Missouri-Kansas City School of Medicine, 2411 Holmes Street, Kansas City, MO 64108 USA; 3 Division of Rheumatology, Children’s Mercy Hospital, 2401 Gillham Road, Kansas City, MO 64108 USA

**Keywords:** Temporomandibular joint, Joint replacement; juvenile idiopathic arthritis, Therapy

## Abstract

**Background:**

Temporomandibular joint (TMJ) arthritis and involvement is commonly seen in Juvenile Idiopathic Arthritis (JIA). Therapy includes conservative measures, but also includes intraarticular corticosteroid injections (IASI) and systemic immunosuppressive therapy. Despite aggressive medical therapy, some patients develop arthritic changes and frank TMJ ankylosis that can result in persistent pain and limitation in range of motion (ROM). A surgical option is prosthetic TMJ replacement with concurrent correction of dentofacial deformities, which can be performed simultaneously. The objective of this study was to evaluate the outcomes of prosthetic TMJ replacement in a cohort of adolescent females with JIA and severe TMJ involvement.

**Methods:**

This is a retrospective case series that took place at one tertiary care center. Patients with a diagnosis of JIA who also underwent alloplastic TMJ replacement were identified through electronic medical record system (EMR) and reviewed. Chart review included analysis of all documents in the EMR, including demographic data, JIA history, surgical complications, ROM of TMJ measured by maximal incisal opening in millimeters (mm) and TMJ pain scores (4-point Likert scale: none, mild, moderate, severe) obtained pre- and postoperatively.

**Results:**

Five female patients, ages 15–17 year when TMJ replacement was performed, had nine total joints replaced with a post-operative follow-up period of 12–30 months. All patients had polyarticular, seronegative JIA and were treated with IASI and multiple immunosuppressive therapies without resolution of TMJ symptoms. One patient had bilateral TMJ ankylosis. Three of the five patients demonstrated significant dentofacial deformities, and all underwent simultaneous or staged orthognathic surgery. All patients had improvement in TMJ pain with most (80%) reporting no pain, and all had similar or improved ROM of their TMJ postoperatively. There was one delayed postoperative infection with Cutibacterium Acnes that presented 15 months after surgery and required removal and reimplantation of prosthesis.

**Conclusion:**

The sequelae of TMJ arthritis and involvement from JIA in the adolescent population can be difficult to treat. Current medical therapy can be successful, however, in select cases that develop chronic changes in the TMJ despite extensive medical therapy, early results show that prosthetic joint replacement maybe a reasonable surgical option. With prosthetic joint replacement pain levels were reduced and range of motion was maintained or improved for all patients.

## Background

Temporomandibular joint (TMJ) arthritis is present in 40–96% of children with Juvenile Idiopathic Arthritis (JIA) [1]. TMJ arthritis can present in all subtypes of JIA, however, appears more prevalent in extended oligoarticular and polyarticular rheumatoid factor negative subtypes [2]. The diagnosis can be challenging as physical exam findings may be absent initially, and become more noticeable later in the disease course with decrease in mouth opening, lateral deviation, and pain with motion [3]. TMJ involvement can be characterized by magnetic resonance imaging (MRI), however, patients may present with a range of clinical signs and symptoms that do not always correlate with radiographic findings [4–6]. Symptoms can vary widely for those with TMJ involvement and some present with combination of pain, limitation in range of motion (ROM), progressive mandibular retrognathia or asymmetry, and imaging ranging from condylar resorption to severe arthritic changes, even TMJ ankylosis [7]. Therapy for TMJ arthritis and involvement can vary broadly from conservative measures such as splint therapy, to arthrocentesis and/or arthroscopy [8, 9], with or without intraarticular steroid injection (IASI) [8, 9], to more complex medical therapies such as systemic immunosuppression (biologic and synthetic disease modifying antirheumatic drugs [DMARDs]). These therapies can be effective for managing patients with TMJ pain and limitation in ROM, however, despite conservative measures in addition to aggressive medical therapy, a small number of patients will require surgical intervention to treat severe TMJ involvement and progressive dentofacial deformities [10]. In certain situations, TMJ replacement may be warranted and can be combined with orthognathic surgery to achieve the best esthetic and functional outcome [11–13]. In adults, prosthetic replacement has become the preferred method of reconstruction to treat the TMJ that has been affected by severe arthritis [14]. However, there is limited data on prosthetic TMJ replacement in children with severe TMJ involvement [13, 15, 16]. Our objective was to evaluate the outcomes of prosthetic TMJ replacement in a cohort of adolescent females with JIA and severe TMJ involvement.

## Methods

In a retrospective case series at Children’s Mercy Kansas City, a tertiary care Children’s hospital, five patients were identified through the electronic medical records (EMR) from January 1, 2017 to December 31, 2018. Patients were identified if they had a diagnosis of JIA and underwent TMJ replacement with either unilateral or bilateral prosthetic joints. Patients were included in the review if they were aged between 12 years and 18 years and met the Edmonton 2001 International League of Associations for Rheumatology (ILAR) criteria for JIA [17]. They were excluded if they either did not have JIA or did not undergo surgery for a prosthetic joint replacement of the TMJ. Individual charts were manually reviewed to confirm JIA diagnosis and TMJ prosthetic joint replacement. Chart review included analysis of all documents included in the EMR, including demographic data, JIA subtype, imaging and laboratory studies, treatment (medical and surgical), surgical complications, and response to treatment. Maximal incisal opening was measured in millimeters (mm) to assess ROM of TMJ, and TMJ pain perception was evaluated using a 4-point visual analog scale (none, mild, moderate, severe) before and after replacement (latest follow-up). Preoperative and postoperative periods were defined by at least 3 months prior to and 3 months after TMJ prosthetic placement.

This study was approved by the institutional review board at Children’s Mercy Kansas City and is in accordance with the ethical standards established in the 1964 Declaration of Helsinki. Formal consent was not required for this type of study, but formal consent was obtained for use of patient pictures.

## Results

**Fig. 1:**
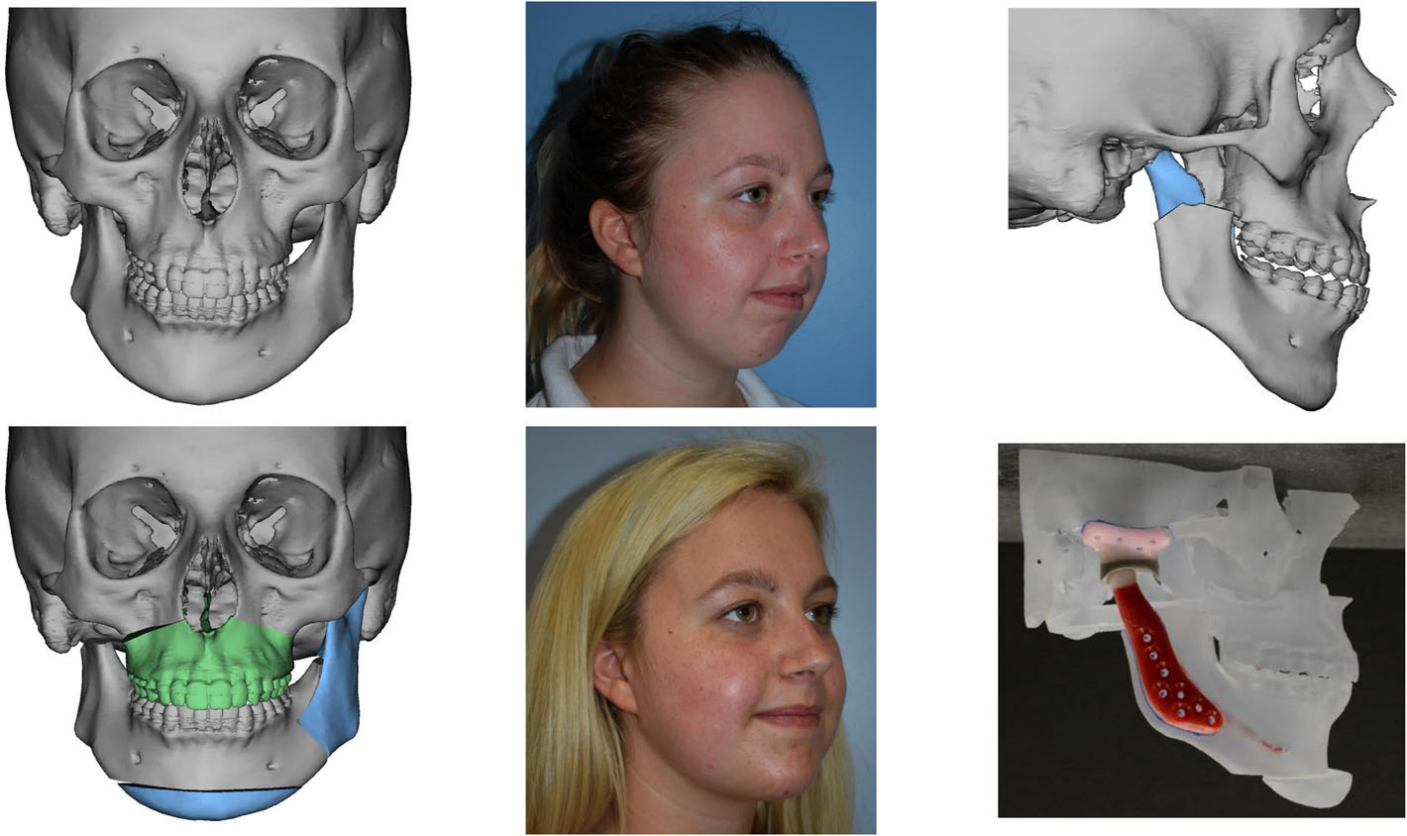
Pre and postoperative images of patient with mandibular asymmetry/deficiency and right TMJ involvement who underwent simultaneous orthognathic surgery with right TMJ replacement. Simulation of orthognathic surgery shown, including Le fort 1 osteotomy, left sagittal split osteotomy, genioplasty, and right condylectomy. Wax up of TMJ prosthesis shown in the patient’s newly simulated jaw position

A total of five patients met inclusion criteria; all were female with an average age of 9.0 years (range 1.2 to 14.4 years) at JIA diagnosis, 13.5 years (range 10.3 to 16.3 years) at age of TMJ arthritis diagnosis, and 16.9 years (range 15.3 to 17.9 years) at age of TMJ prosthetic replacement. All patients had polyarticular, seronegative JIA. All patients had MRI with contrast that showed abnormalities suggestive of TMJ involvement prior to surgery, which included: synovitis, erosion, marrow edema, effusion, sclerosis, flattened condyles, disc displacement, thinning and absence. One of the patients exhibited overt bilateral TMJ ankylosis with severe limitation in maximal incisal opening (15 mm) preoperatively. Maximal incisal opening recorded immediately preoperative averaged 33 mm and ranged from 15 mm to 45 mm and was similar or improved postoperatively with an average of 37 mm at latest follow up (range 30 mm to 45 mm) (Table 1). All five patients had an element of mandibular hypoplasia, and three of the five patients demonstrated mandibular hypoplasia significant enough to consider orthognathic surgery. One patient (shown in Fig. 1.) had mandibular hypoplasia with asymmetry, the right condyle being more severely affected. All five patients reported TMJ pain preoperatively with four reporting severe pain with TMJ motion and one reporting mild pain. Two of the patients had chronic pain syndrome while an additional patient was suspected to have chronic pain syndrome, however, at the postoperative evaluation only one patient had mild TMJ pain (which later resolved) while the rest had no TMJ pain (Table 1). Chronic widespread pain scores were not collected since TMJ was the only joint of interest in this case series. All patients have remained without TMJ pain throughout the postoperative period.

**Table 1:** Demographics and Outcomes

ID	Age^a^	Diagnosis^b^	TMJ^c^ Replacement Laterality	Facial Diagnosis	Pain level^d^	Maximal incisal opening (mm)	Pain level^d^	Maximal incisal opening (mm)	Follow-up^e^
					**Preoperative**		**Postoperative**		
1	16	Polyarticular ANA+	Bilateral	Mandibular hypoplasia	Severe	35	None	35	30
2	17	Polyarticular ANA-	Bilateral	Mandibular hypoplasia	Severe	30	mild	35	29
3	17	Polyarticular ANA-	Right	Mandibular hypoplasia/asymmetry	Severe	45	None	45	15
4	15	Polyarticular ANA-	Bilateral	Mandibular hypoplasia/ankylosis	Mild	15	None	30	12
5	17	Polyarticular ANA-	Bilateral	Mandibular hypoplasia	Severe	40	None	40	12

^a^Age in years

^b^Juvenile idiopathic arthritis subtype diagnosis

^c^Temporomandibular joint

^d^Pain level is a 4-point Likert scale (none, mild, moderate, severe)

^e^Follow-up in months

Maximal incisal opening was recorded immediately preoperatively and at latest follow-up visit (at least 6 months from surgery)

For therapy, all patients were initially referred to primary dentist for occlusal stabilization splits, however, there was minimal degree of TMJ symptom reduction and difficulty with compliance in dental follow-up and split use. Patients also received at least one arthrocentesis with steroid injection (most patients within 6 months of TMJ replacement) with one patient having multiple injections (Table 2). There was an average of 2.8 years (range 0.3 to 6.3 years) from JIA diagnosis to introduction of the first biologic DMARD for treatment of JIA. All patients had history of biologic and synthetic DMARD therapies for JIA treatment prior to preoperative period, but during course of treatment (Table 2) and at diagnosis of TMJ arthritis one patient was on no systemic therapy, three patients were on a synthetic DMARD in combination with biologic DMARD, while one was on combination synthetic DMARD therapy. At diagnosis of TMJ arthritis, all patients had IASI of the affected TMJ joint, while one had addition of biologic DMARD and two had change in their biologic DMARD (Table 2). At diagnosis of TMJ arthritis by MRI with contrast three patients clinically had TMJ arthritis only, while the other two patients had TMJ arthritis and other joints with active arthritis (one with active arthritis in one knee and the other with active arthritis in one wrist). There was an average of 3.4 years between initial evidence of TMJ arthritis and surgical replacement. During the perioperative period, all patients had their biologic and synthetic DMARDs held for at least one dosing cycle prior to TMJ replacement. Biologic and synthetic DMARD therapy was resumed at least 14 days after TMJ replacement. By the postoperative period, all patients were on combination biologic and synthetic DMARD therapy.

**Table 2:** Medical and Surgical Therapies

ID	Intraarticular Steroid injection	Orthognathic Surgery	Concurrent procedures	Medical therapy	
				Preoperative	Postoperative
1	Yes	No	No	Methotrexate^**c**^ Abatacept^c^ Etanercept Adalimumab Infliximab Tocilizumab	Methotrexate^c^ Infliximab^c^
2	Yes	Le Fort I, bilateral sagittal splits (staged)	Submental liposuction	Methotrexate^c^ Hydroxychloroquine^c^ Adalimumab^c^ Etanercept Infliximab	Methotrexate^c^ Abatacept^c^
3	Yes	Le Fort I, left sagittal split, genioplasty (simultaneous)	Submental liposuction	Hydroxychloroquine^c^ Sulfasalazine^c^ Methotrexate^c^ Abatacept^c^ Etanercept Adalimumab Infliximab	Leflunomide^c^ Adalimumab^c^ Tofacitinib Sulfasalazine
4	Yes^a^	Genioplasty (simultaneous)	No	Methotrexate^c^ Etanercept^c^ Adalimumab	Methotrexate^c^ Adalimumab^c^
5	Yes^b^	No	No	None^c^ Etanercept Infliximab Adalimumab	Methotrexate^c^ Infliximab^c^

^a^Arthrocentesis with steroid injection performed at outside institution

^b^Patient had arthrocentesis with steroid injection three times pre-operatively

^c^Denotes perioperative therapy that patient was on 3 months preoperatively and 3 months postoperatively

There were nine total joints replaced using the TMJ concepts (Ventura, CA) custom fitted prosthesis. Patients had a post-operative follow-up that ranged from 12 to 30 months. There was one delayed postoperative infection that presented 1 year after surgery. The patient presented with isolated TMJ pain without systemic signs of infection. There was no redness, swelling, fever, or TMJ limitation. Further investigation revealed a normal CT scan without any overt signs of infection or implant malfunction. Surgical exploration was performed and did not reveal any overt signs of infection or implant malfunction. The TMJ fossa was cultured and grew Cutibacterium Acnes. An antibiotic spacer was placed, and oral antibiotics initiated for a four-month period. After the period of antibiotics, the TMJ prosthesis was replaced. Recurrent or additional infections have not occurred in this patient or others since that time. Other adverse outcomes included one patient with right-sided temporal branch facial weakness, and one patient with marginal mandibular weakness, both of which resolved over a two-month period.

## Discussion

TMJ arthritis and involvement is present in many children with JIA and can be difficult to monitor and treat. While conservative measures and medical therapies are most used and appropriate [1], a definitive surgical option may be needed for a patient suffering from chronic TMJ involvement with pain, limited ROM, and history of maximized conservative therapies such as splints, arthrocentesis, arthroscopy and IASI and aggressive systemic therapies such as synthetic and biologic DMARDs. The decision to perform prosthetic TMJ replacement in the adolescent with JIA is a difficult one, and there is no consensus among maxillofacial surgeons regarding the preferred treatment [18, 19]. Certainly, it must be a consideration in patients with severe TMJ symptoms, involvement, deformity and dysfunction who had no improvement with multiple medical and conservative therapies, and perhaps the preferred treatment in cases of TMJ ankylosis [13] or other dentofacial deformities. Despite encouraging long term results [20, 21], it is likely a prosthetic would need to be replaced during adulthood due to wear of the prosthesis over time. The time period for revision or re-replacement is currently unknown. Additionally, there is an inherent risk of infection of any prosthetic appliance, which is more concerning in a population that is being treated with immunosuppressant medications. The risks of TMJ replacement should not be taken lightly, but the risks are often outweighed by the benefit of effective pain relief with a stable joint and preserved ROM, all of which was accomplished in this cohort.

Evidence remains unclear on the efficacy of systemic therapy for treatment of TMJ arthritis as part of JIA, and that TMJ arthritis may behave differently compared to other joint disease seen with JIA [1]. Local therapies such as IASI have been shown to decrease inflammation in the TMJ, however, serious adverse effects of heterotrophic bone formation and condylar resorption have also been seen with IASIs [22], causing some to limit the use or repeat use of IASI. Others support maximizing systemic medical therapy as an alternative or in conjunction with IASI based on the severity of disease [1]. All the patients in this study had IASI and aggressive maximization of their systemic therapies with use of different combinations of biologic and synthetic DMARDs at time of TMJ arthritis diagnosis and prior to surgery, but despite those efforts, developed chronic, worsening TMJ pain due to TMJ involvement and damage and ultimately opted for surgical intervention. There was an average of 2.8 year between JIA diagnosis and introduction of first biologic DMARD, which may indicate that earlier introduction of biologic DMARD is necessary to minimize TMJ damage and symptoms, however, one patient did have biologic DMARD introduced 4 months after JIA diagnosis and still required TMJ replacement.

Two of the patients had a diagnosis of chronic pain syndrome, and a third had concerning features suggestive of chronic pain syndrome, which is associated with chronic, widespread pain, and commonly involve other temporomandibular disorders. There is evidence that orofacial pain in adolescents with JIA can be associated with stress, depression and catastrophizing [23], and therefore can be difficult to discern TMJ involvement versus TMJ disorder. For the patients in this cohort, all had isolated worsening of TMJ symptoms with MRI evidence of chronic damage which is more suggestive of TMJ involvement. It should be noted that continuous abnormalities may be seen on repeat MRI due to damage from mechanical changes that are secondary to the initial chronic inflammatory changes. This is likely the cause of the isolated worsening TMJ pain despite aggressive conservative and systemic therapy with biologic and synthetic DMARDs. Additionally, with TMJ replacement all patients had resolution of TMJ pain, despite three of the patients continuing to have chronic pain in locations outside the TMJ. We recommend diligence in determining the exact cause of TMJ symptoms prior to surgery, but isolated worsening of TMJ symptoms along with MRI evidence of TMJ damage maybe an early sign that surgical intervention should be considered.

Many patients with JIA have TMJ involvement with dentofacial deformities that can result in pain and limited ROM. This can result as either a direct consequence of their condition or due to pre-existing conditions, such as mandibular retrognathia or mandibular asymmetry [24]. Elective orthognathic surgery to correct significant malocclusion in a patient with JIA is typically deferred for orthodontic camouflage so as not to affect joint health, but the esthetic and psychologic benefits of corrective jaw surgery cannot be overlooked [25]. A potential benefit in performing TMJ replacement is that concurrent orthognathic surgery is possible. The decision to perform orthognathic surgery simultaneous with joint replacement is one based on severity of deformity and patient preference. By no means is simultaneous orthognathic surgery obligatory with joint replacement. With routine employment of three-dimensional virtual surgical planning, TMJ replacement and orthognathic surgery can be planned and performed simultaneously (Fig. 1). Orthodontic treatment to align the dental arches prior to or immediately after combined joint replacement/orthognathic surgery is warranted. Arguably, any JIA patient with TMJ involvement, and certainly any patient who is a candidate for joint replacement, should be evaluated by an orthodontist to address concurrent malocclusion.

A potential complication after joint replacement is infection, and one patient in this series developed infection 1 year after surgery without typical signs of infection. The patient described periodic swelling and pain of the cheek over the TMJ that was not clinically noticeable. It is possible that the patient’s immunosuppressive therapies altered the presentation of the infection, however, it is unclear. In an adult retrospective review of total joint prostheses, 8 of 579 protheses were noted to be infected. It was found that acute infection (within 30 days of surgery) was more frequent (5/8) than delayed infections (31 days to 6 years; 3/8). A treatment strategy for acute infections included a protocol of debridement, irrigating catheters, and intravenous antibiotics to salvage the prosthesis, while delayed infections usually require removal and replacement of the prosthesis [26]. In the delayed infections group, one of three patients grew Cutibacterium acnes [26], which was the same bacterium isolated in our case. Additionally, the patient in our case also required removal and replacement of the prosthesis.

It is currently unclear how immunosuppressant therapies used for JIA may impact TMJ infections after prosthesis placement. Most literature is focused on infection related orthopedic surgery, but not specifically regarding the maxillofacial complex. Methotrexate, when continued or stopped prior surgery, was not shown to increase early complications after orthopedic surgery in a randomized trial [27], while the influence on infection rates has been debated [28]. A retrospective study [29] examined the risk of postoperative infection at the site of various orthopedic surgeries in patients with rheumatoid arthritis who were treated with immunosuppressants (mostly biologic and synthetic DMARDs), and found 0.8% (of 50,359 surgical cases) had postoperative infections. It was noted that the risk of infection significantly increased in patients taking multiple synthetic DMARDs or combination therapy with biologic and synthetic DMARDs. In this case series, all patients had their biologic and synthetic DMARDs held before and after surgery, however, the American College of Rheumatology does offer perioperative therapy guidance for adult patients with rheumatic disease undergoing elective surgery, and suggests that synthetic DMARDs (specifically methotrexate, leflunomide, hydroxychloroquine and sulfasalazine) may be continued through surgery. Further, biologic DMARDs should be held for at least one dosing cycle with planned surgery at the end of the dosing cycle and may be resumed once wounds show evidence of healing (approximately 14 days) [30].

The limitations of this study include the small sample size and retrospective design, which precludes more sophisticated data analysis including subgroup analysis of patients based on initial presentation and therapy. However, this is the largest cohort of JIA patients with TMJ prosthesis reported to date. Future studies in a larger population will allow for more detailed exploration of outcomes and adverse events.

## Conclusion

TMJ arthritis is present in many children with JIA, and while conservative and aggressive systemic medical therapies such as biologics and synthetic DMARDs are preferred, some that are refractory to extensive medical therapy may benefit from prosthetic joint replacement, which can improve pain and maintain maximal incisal opening. Infections are a potential complication and risk factor in an immunocompromised patient undergoing surgery for a prosthetic joint implant. Further studies are needed with greater numbers of patients, and with longer follow up to assess pain, ROM and adverse events.

## Data Availability

The datasets used and analyzed during the current study are available from the corresponding author on reasonable request.

## Abbreviations

ANA: Anti-nuclear antigen
DMARD: Disease modifying anti-rheumatic drug
EMR: Electronic medical record system
IASI: Intraarticular corticosteroid injections
ILAR: International League of Associations for Rheumatology
JIA: Juvenile Idiopathic Arthritis
mm: Millimeters
ROM: Range of motion
TMJ: Temporomandibular joint
